# Modified *ppk* Expression Alters Temporal Transcriptional and Phosphorus–Nitrogen Responses to Phosphate Fluctuation in *Synechococcus* sp. PCC 7002

**DOI:** 10.3390/microorganisms14091997

**Published:** 2026-09-09

**Authors:** Sijia Shen, Enci Ye, Tao Li, Zi Ye

**Affiliations:** 1 Institute of Hydrobiology, Chinese Academy of Sciences, Wuhan 430072, China; shensijia@ihb.ac.cn (S.S.); yeenci@ihb.ac.cn (E.Y.); litao@ihb.ac.cn (T.L.); 2 University of Chinese Academy of Sciences, Beijing 100049, China; 3 Key Laboratory of Algal Biology, Chinese Academy of Sciences, Wuhan 430072, China; 4 State Key Laboratory of Lake and Watershed Science for Water Security, Institute of Hydrobiology, Chinese Academy of Sciences, Wuhan 430072, China

**Keywords:** *Synechococcus* sp. PCC 7002, polyphosphate kinase, phosphate fluctuation, regulatory refactoring, time-resolved transcriptomics, phosphorus–nitrogen coordination

## Abstract

Phosphate availability fluctuates widely in aquatic environments, yet how engineering the regulatory context of polyphosphate kinase affects cyanobacterial responses to such fluctuations remains unclear. Here, the wild-type strain of *Synechococcus* sp. PCC 7002 and Dppk, a modified *ppk*-expression strain derived from the WT background, were subjected to phosphate starvation followed by phosphate resupply. Dense RT-qPCR sampling was used to resolve rapid *ppk*-expression dynamics, and time-resolved RNA sequencing was performed at seven stages with four biological replicates per condition. WT displayed rapid, stage-dependent changes in *ppk* expression, including a pronounced late induction after phosphate resupply, whereas Dppk showed a more moderate temporal pattern. Transcriptome-wide analyses further revealed distinct basal states and response trajectories between the strains. Functional analyses linked these differences to photosynthesis-related processes, phosphate transport, and polyphosphate metabolism. These findings indicate that refactoring *ppk* expression alters both target-gene kinetics and the temporal organization of the broader phosphate-responsive transcriptional network.

## 1. Introduction

Phosphorus is an essential yet frequently limiting and highly dynamic resource in aquatic environments [1,2,3]. It is incorporated into nucleic acids, ATP, phospholipids, and numerous phosphorylated metabolic intermediates, while the concentration of bioavailable inorganic phosphate can vary substantially across habitats and over time [3,4,5]. In many aquatic systems, phosphate is supplied intermittently as short-lived pulses against a background of scarcity [6]. Cyanobacteria must therefore manage repeated transitions between phosphate deprivation and renewed availability rather than acclimate only to a stable nutrient condition. Their adaptive repertoire includes high-affinity phosphate transport, extracellular phosphorus scavenging, utilization of alternative phosphorus sources, and intracellular storage as polyphosphate (polyP) [7,8,9]. Natural cyanobacterial communities likewise adjust cellular phosphorus quotas and polyP pools in response to changing phosphate availability [10].

The cyanobacteria’s response to phosphate limitation is controlled by a broad regulatory network rather than by a single transport pathway. In *Synechocystis* sp. PCC 6803 (hereafter PCC 6803), the SphS–SphR two-component system regulates major phosphate-responsive genes, including high-affinity phosphate transport and extracellular phosphorus-scavenging functions [11]. The PstSCAB transporter plays a central role in phosphate acquisition, and the two Pst systems of PCC 6803 display distinct transport properties that may broaden the range of phosphate concentrations over which uptake can occur [12]. However, phosphate acclimation extends beyond transport. Transcriptomic studies in *Synechococcus* sp. WH8102 and Prochlorococcus MED4 have shown that the genes induced during early phosphate stress differ partly from those maintained during prolonged deprivation, and that phosphate starvation and chronic limitation produce distinguishable transcriptional states [13,14]. These findings emphasize the need for time-resolved analyses that separate rapid responses to phosphate removal from later starvation and recovery phases.

PolyP connects phosphate acquisition with intracellular storage and subsequent remobilization. Its synthesis is primarily catalyzed by polyphosphate kinase (PPK), whereas exopolyphosphatase contributes to polymer degradation and phosphate release. Cyanobacterial polyP pools can accumulate rapidly when phosphate-deprived cells encounter renewed phosphate and then decline as growth and biosynthetic demand resume [15,16,17]. Recent genetic evidence further indicates that PPK-dependent polyP accumulation contributes to acclimation during nutrient loss, supporting a role for polyP metabolism in broader stress-response physiology rather than as a passive storage process [18]. Nevertheless, phosphate transport, *ppk* transcription, and polyP abundance represent related but non-equivalent layers of phosphorus metabolism. The regulatory architecture surrounding *ppk* may therefore influence not only transcript abundance but also the timing with which phosphate signals are propagated through the cellular network.

This issue is particularly relevant when *ppk* is expressed outside its native regulatory context. Conventional overexpression mainly changes gene dosage or promoter strength, whereas regulatory refactoring alters how an engineered unit is connected to endogenous control systems [19]. *Synechococcus* sp. PCC 7002 (hereafter PCC 7002) is suitable for examining this distinction because it is genetically tractable and has established modular expression tools [20,21,22]. Recent work has further shown that PCC 7002 coordinates phosphate transport with photosynthetic, biosynthetic, and central metabolic functions during phosphate starvation and recovery [23]. It remains unclear, however, whether refactoring *ppk* merely alters the abundance of the target transcript or also reshapes the timing and composition of the wider phosphate-responsive network.

Potential network-level effects may extend beyond phosphorus homeostasis because phosphorus, nitrogen, carbon, and cellular energy metabolism are physiologically coupled. In cyanobacteria, intracellular 2-oxoglutarate reflects the balance between carbon supply and nitrogen assimilation and regulates the global nitrogen transcription factor NtcA [24]. The signal transduction protein PII, together with PipX, integrates 2-oxoglutarate and adenylate status with NtcA-dependent regulation, while PII also controls the uptake of ammonium, nitrate/nitrite, and urea [25,26]. Notably, the experimentally defined NtcA regulon in PCC 6803 includes pstS, which encodes a phosphate-binding component of the Pst transporter [27]. These links provide a mechanistic basis for coordinated phosphorus–nitrogen responses, but it is unknown whether changing the regulatory architecture of a polyP-related gene alters overall P-N coordination or selectively redistributes individual relationships between phosphorus- and nitrogen-responsive modules.

Accordingly, this study aimed to characterize how the temporal transcriptional response to phosphate fluctuation differs between the WT strain of PCC 7002 and Dppk, an engineered strain with a modified *ppk* expression architecture. In Dppk, a P*_cpcBA_*-driven *ppk* copy was retained after replacement of the endogenous *ppk* locus, thereby separating *ppk* expression from part of its native regulatory context. Cells were subjected to phosphate starvation followed by phosphate resupply. A densely sampled RT-qPCR series was used to resolve rapid *ppk*-expression dynamics, while time-resolved RNA sequencing captured the establishment and recovery of broader transcriptional states. We examined target-gene kinetics, global transcriptomic trajectories, phosphate-homeostasis and photosynthesis-related programs, and the coordination of phosphorus- and nitrogen-associated modules. We hypothesized that Dppk would exhibit a distinct temporal response to phosphate fluctuation relative to WT, including altered *ppk* expression dynamics and broader differences in phosphate-responsive gene expression.

## 2. Materials and Methods

**Cyanobacterial Strains and Culture Conditions**

The wild-type (WT) strain of PCC 7002 was maintained in our laboratory, and Dppk, a refactored *ppk*-expression strain, was constructed from the WT background. Cells were routinely cultivated in A+ medium [28,29], the composition of which is provided in Supplementary Table S1. Liquid cultures were grown at 30 °C under continuous illumination of 40 μmol photons m^−2^ s^−1^ with shaking at 110 rpm. For cultivation on solid medium, A+ medium was supplemented with 1.2% (*w*/*v*) agar, and plates were incubated at 30 °C under continuous illumination of 30 μmol photons m^−2^ s^−1^. Media were sterilized by autoclaving, whereas heat-labile stock solutions were sterilized by membrane filtration.

For routine maintenance of Dppk, kanamycin and spectinomycin were added at 50 μg mL^−1^ each.

**Construction and Verification of the Dppk Strain**

The *ppk* coding sequence of PCC 7002 (IMG gene ID 2708940341) was amplified from genomic DNA and cloned downstream of the strong P*_cpcBA_* promoter in the pAQ1EX-PCP vector. The resulting *ppk* expression cassette was integrated into the endogenous pAQ1 plasmid of PCC 7002 by homologous recombination to generate the intermediate overexpression strain *ppk*-OE. The native *ppk* locus was subsequently replaced with a kanamycin-resistance cassette through double homologous recombination, yielding the Dppk strain while retaining the promoter-driven *ppk* copy in pAQ1. The recombinant vector was verified by restriction digestion, and integration of the expression cassette, replacement of the native *ppk* locus, and complete segregation of the resulting strain were confirmed by diagnostic PCR and Sanger sequencing. Primers used for strain construction and verification are listed in Supplementary Table S2.

**Phosphate Starvation and Resupply**

WT and Dppk cultures were grown independently to logarithmic phase in phosphate-replete A+ medium. An aliquot of each culture was collected immediately before phosphate removal and designated as the normal-phosphate control (N). The remaining cells were harvested by centrifugation at 5000× *g* for 5 min, washed three times with phosphate-free A+ medium, and resuspended in phosphate-free A+ medium to an initial OD_730_ of 0.4.

Samples were collected from the same four cultures at 30 min, 24 h, and 48 h after transfer to phosphate-free medium and were designated P0–30 min, P0–24 h, and P0–48 h, respectively. After 48 h of phosphate starvation, K_2_HPO_4_ was added to a final concentration of 2.87 mM, corresponding to ten times the phosphate concentration of standard A+ medium. This concentration was selected to establish a defined high-phosphate condition and to maintain phosphate availability throughout the subsequent 48 h sampling period. Cell pellets were immediately frozen in liquid nitrogen and stored at −80 °C until RNA extraction.

For the densely sampled RT-qPCR time course, samples were collected at 1, 2, 5, 10, 20, and 30 min and at 1, 2, 3, and 6 h after phosphate removal and resupply. For Western blot verification, samples were collected under normal phosphate conditions, after 6 h of phosphate starvation, and 3 h after phosphate resupply.

**RT-qPCR Analysis**

Total RNA was extracted from logarithmic-phase cyanobacterial cells using the PureLink™ RNA Mini Kit (Invitrogen, Carlsbad, CA, USA) according to the manufacturer’s instructions, with minor modifications for cell disruption. Briefly, cells were collected by centrifugation at 5000× *g* for 5 min at 4 °C and resuspended in 100 μL of lysozyme solution. After the addition of 0.5 μL of 10% SDS, the samples were vortexed and incubated at room temperature for 20 min. Lysis buffer containing 2-mercaptoethanol was then added, and the lysates were homogenized before column purification. Total RNA was eluted with 30 μL of RNase-free water. RNA concentration and purity were assessed using a NanoDrop spectrophotometer (Thermo Fisher Scientific, Waltham, MA, USA), and the samples were stored at −80 °C until further analysis.

Genomic DNA was removed, and first-strand cDNA was synthesized using the PrimeScript™ FAST RT Reagent Kit with gDNA Eraser (TaKaRa, Kusatsu, Japan) according to the manufacturer’s instructions. RT-qPCR was performed using TB Green^®^ Premix Ex Taq™ II FAST qPCR (TaKaRa, Japan) on a LightCycler^®^ 480 II system (Roche, Basel, Switzerland). The 16S rRNA gene was used as the internal reference gene. It was selected based on previous evaluations of reference gene suitability in cyanobacteria and its application in PCC 7002-related studies [30]. The relative *ppk* expression was calculated using the (2^−ΔΔ Ct^) method. Primer sequences are listed in Supplementary Table S2.

**Protein Extraction and Western blot Analysis**

A rabbit polyclonal antibody against PPK was custom-produced by ABclonal (Wuhan, China). Total protein was extracted using the Minute™ Total Protein Extraction Kit for Microorganisms with Thick Cell Walls (Invent Biotechnologies, Plymouth, MN, USA) according to the manufacturer’s instructions. Protein concentrations were determined using a BCA Protein Assay Kit (Beyotime, Shanghai, China), and extracts were stored at −80 °C until analysis.

Equal amounts of total protein were mixed with 5× loading buffer and denatured at 95 °C for 10 min. Samples were separated on SDS-PAGE gels in HEPES running buffer at 80 V for approximately 90 min. A PageRuler™ Prestained Protein Ladder (Thermo Scientific, Waltham, MA, USA) was used as the molecular-mass marker. Proteins were transferred to PVDF membranes in precooled transfer buffer at a constant current of 150 mA for 1 h.

Membranes were blocked overnight at 4 °C in PBST containing 5% (*w*/*v*) non-fat dry milk and incubated with the custom anti-PPK primary antibody (1:5000) for 2 h at room temperature. After three 5-min washes with PBST, membranes were incubated with HRP-conjugated anti-rabbit IgG (H + L) secondary antibody (Promega, Madison, WI, USA; 1:10,000) for 1 h at room temperature. Following three additional washes with PBST, signals were developed using an enhanced ECL chemiluminescent substrate kit (BioSharp, Hefei, China) and captured with a ChemiScope 6100 imaging system (Clinx Science Instruments, Shanghai, China). The Western blot was used for qualitative verification of protein abundance. No lane-specific loading control normalization or densitometry analysis was performed.

**RNA Extraction, Library Preparation, and Sequencing**

Total RNA was extracted from frozen cell pellets using TRIzol reagent (Invitrogen, Carlsbad, CA, USA) according to the manufacturer’s instructions, following the principle [31]. Cells were collected by centrifugation at 4000–5000× *g* for 5 min, immediately frozen in liquid nitrogen, and stored at −80 °C until RNA extraction. Each treatment included four biological replicates. All WT and Dppk samples were processed and sequenced within the same experimental batch.

RNA concentration and purity were assessed using a NanoDrop One spectrophotometer (Thermo Fisher Scientific, Waltham, MA, USA), and RNA integrity was evaluated using an Agilent 4200 TapeStation system (Agilent Technologies, Santa Clara, CA, USA). Ribosomal RNA was removed from qualified RNA samples using the Epicentre Ribo-Zero rRNA Removal Kit. Strand-specific RNA-seq libraries were constructed using the NEBNext^®^ Ultra II™ Directional RNA Library Prep Kit for Illumina (New England Biolabs, Ipswich, MA, USA). Briefly, RNA was fragmented and reverse-transcribed using random hexamer primers, followed by second-strand cDNA synthesis with dUTP incorporation. After end repair, A-tailing, adaptor ligation, USER enzyme treatment, size selection, PCR amplification, and purification with AMPure XP beads, the final libraries were obtained. Qualified libraries were sequenced on an Illumina NovaSeq X Plus platform (Illumina, Inc., San Diego, CA, USA) to generate 150-bp paired-end reads.

**RNA-seq Data Processing and Expression Quantification**

Raw FASTQ reads were processed using in-house Perl scripts and Trimmomatic to remove adapter sequences and trim low-quality bases [32]. Reads shorter than 50 nt and unpaired reads were discarded. Q20, Q30, and GC-content statistics were calculated for the retained clean reads. All downstream analyses were performed using the quality-filtered paired-end reads.

The reference genome index was constructed using Bowtie2 v2.2.6 [33], and clean reads were aligned to the PCC 7002 reference genome (NCBI RefSeq assembly GCF_000019485.1; ASM1948v1). Gene-level read counts were generated using HTSeq-count v0.6.0 and the corresponding RefSeq genome annotation [34]. FPKM values were calculated according to mapped fragment counts and annotated gene lengths [35] and were used for expression visualization and heatmap construction. Raw gene-level read counts were used for differential expression analysis.

**Differential Expression and Functional Analyses**

Differential expression analysis was performed using DESeq2 v1.52.0 based on raw gene-level counts [36]. Because the same four biological cultures of each strain were repeatedly sampled across the seven experimental stages, culture identity was included in the statistical design. Within each strain, stage-dependent differential expression was analyzed using a model including culture and stage, with each phosphate-starvation or resupply stage compared with the corresponding normal-phosphate baseline (N). Dppk and WT were also compared at matched stages.

Strain-dependent temporal responses were evaluated using a DESeq2 strain × stage interaction model, with biological replicates nested within strain. The full model (~strain + strain: culture + stage + strain: stage) was compared with a reduced model lacking the interaction term using a likelihood-ratio test. Stage-specific interaction coefficients were subsequently used to assess differences in response between WT and Dppk relative to N.

*p* values were adjusted using the Benjamini–Hochberg procedure [37]. Conventional DEGs were defined as adjusted *p* < 0.05 and an absolute log_2_ fold change ≥ 1, whereas interaction significance was defined as adjusted *p* < 0.05. Variance-stabilizing transformation (VST) was applied to the raw count data for downstream multivariate analyses. PCA was performed using the 500 most variable genes, and Pearson sample correlations were calculated from the VST-transformed expression matrix. Response-concordance analysis was based on within-strain log2 fold changes and was used descriptively; statistically significant differences between strains were assessed using the interaction analysis described above.

**Functional Enrichment Analysis**

Gene Ontology (GO) and KEGG pathway over-representation analyses were performed using the enricher function implemented in clusterProfiler v4.20.0 [38]. Custom term-to-gene and term-to-name annotation mappings were constructed for PCC 7002. Genes included in the DESeq2 analysis and possessing the corresponding GO or KEGG annotations were used as the background gene set. Enrichment *p* values were adjusted using the Benjamini–Hochberg procedure, and terms or pathways with an adjusted *p* value < 0.05 were considered significantly enriched. For visualization of GO enrichment, redundancy among significant terms was reduced based on overlap of their contributing gene sets using a Jaccard similarity threshold of 0.70. Representative nonredundant terms were ranked by adjusted *p* value and fold enrichment for display, whereas all significant KEGG pathways were retained. Complete enrichment outputs and contributing gene lists are provided in the Supplementary Data S4 and S5.

**Phosphorus–Nitrogen-Related Transcriptional Analysis**

Phosphorus- and nitrogen-related genes were selected according to their annotated functions in phosphate uptake, polyphosphate metabolism, phosphate-stress response, nitrogen regulation, nitrogen assimilation, and nitrogen transport. Complete gene lists and functional-module assignments are provided in Table S3. For module-level temporal analysis, expression values were standardized gene-wise across the experimental series, and the mean z-score of genes assigned to each module was calculated for each strain–stage group. For the targeted P–N association analysis, Pearson correlation coefficients were calculated separately for WT and Dppk using the 28 sample-level expression profiles per strain (four biological cultures sampled across seven stages), and differences were expressed as Δr = rDppk − rWT. P–N pairs with |Δr| ≥ 0.5 were retained for network visualization. Because repeated samples from the same culture were not statistically independent, the correlation analysis was treated as an exploratory description of transcriptional associations.

**Statistical Analysis**

Unless otherwise stated, data are presented as the mean ± SEM from independent biological replicates. Statistical analyses for RT-qPCR were performed using R (4.6.0). The gene-level read-count matrix, sample metadata, complete different expression results, complete GO and KEGG enrichment results and the underlying gene definitions and numerical outputs used in the targeted P-N analyses are provided in Supplementary Data S1–S6.

## 3. Results

### 3.1. Construction of the Dppk Strain and Phosphate-Fluctuation Experimental Design

To investigate how altered *ppk* regulation affects the response of sp. PCC 7002 to phosphate fluctuation, we constructed the refactored *ppk*-expression strain Dppk. Preliminary attempts to disrupt the native *ppk* locus directly did not yield a recoverable, fully segregated mutant, suggesting that complete loss of *ppk* was not tolerated under the tested conditions. We therefore adopted a two-step strategy in which an additional functional *ppk* copy was introduced before replacement of the native locus.

**Figure 1:**
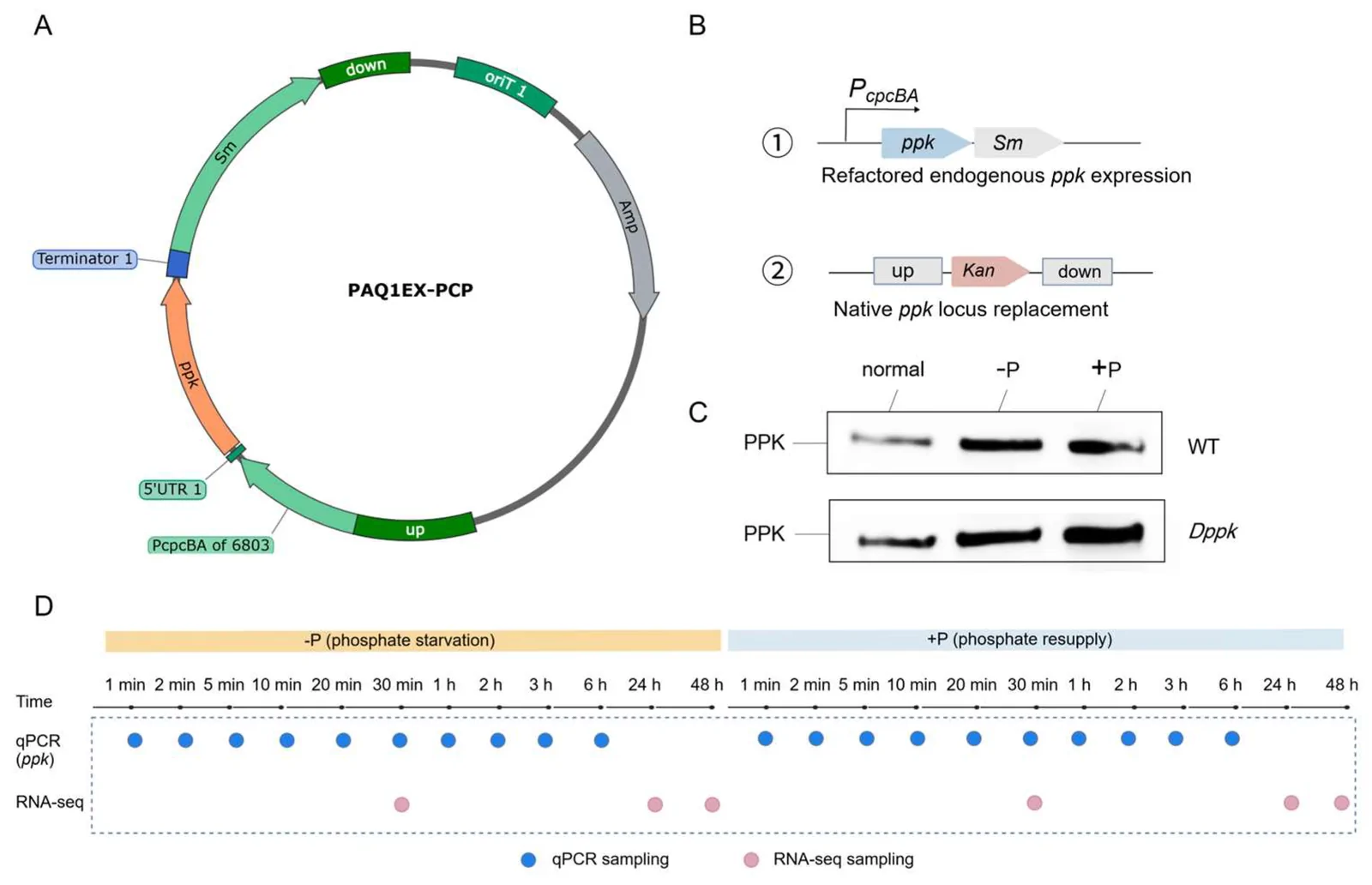
Construction and verification of an engineered Dppk strain and experimental design for phosphate-fluctuation analysis. (**A**) Plasmid map of pAQ1EX-PCP used for *ppk* expression. The *ppk* coding sequence from PCC 7002 was placed downstream of the strong P*_cpcBA_* promoter from PCC 6803. (**B**) Schematic representation of the two-step Dppk construction strategy. The *PcpcBA-ppk* cassette was first introduced into endogenous pAQ1 plasmid to generate the intermediate ppk-OE strain, followed by replacement of the native *ppk* locus with a kanamycin-resistance cassette to obtain Dppk. (**C**) Western blot detection of PPK in WT and Dppk under normal-phosphate, phosphate-starvation, and phosphate-resupply conditions. The blot is presented as qualitive verification of PPK proteins detection. (**D**) Experimental sampling scheme. RT-qPCR samples were collected from 1 min to 6 h under phosphate starvation (−P) and phosphate resupply (+P), whereas RNA-seq samples were collected under normal-phosphate conditions and at 30 min, 24 h, and 48 h after each treatment transition. This figure was created with BioGDP.com [39].

The PCC 7002 *ppk* coding sequence was placed under the control of the strong promoter P*_cpcBA_* in the pAQ1EX-PCP vector. The promoter-driven cassette was first integrated into the pAQ1 to generate the ppk-OE. The native *ppk* locus was then replaced with a kanamycin-resistance cassette, yielding Dppk while retaining the engineered *ppk* copy. Restriction digestion and Sanger sequencing confirmed the correct plasmid assembly, while diagnostic PCR verified the expected engineered loci and complete segregation of Dppk (Figure 1B). Thus, Dppk retained promoter-driven *ppk* expression but lacked the native *ppk* locus and its original regulatory context.

Western blot analysis detected PPK at the expected molecular mass region in both WT and Dppk, providing qualitative protein-level verification of PPK expression (Figure 1C). Under the cultivation conditions used in this study, no obvious differences in culture color, gross cell morphology, or apparent cell size were observed between WT and Dppk. RT-qPCR samples were collected at closely spaced time points from 1 min to 6 h after each phosphate transition, while RNA-seq samples were collected under normal-phosphate conditions and at 30 min, 24 h, and 48 h after phosphate removal and resupply. This design enabled comparison of the early and prolonged transcriptional responses of WT and Dppk to changing phosphate availability.

### 3.2. Engineered ppk Expression Shows Attenuated Temporal Dynamics During Phosphate Transitions

**Figure 2:**
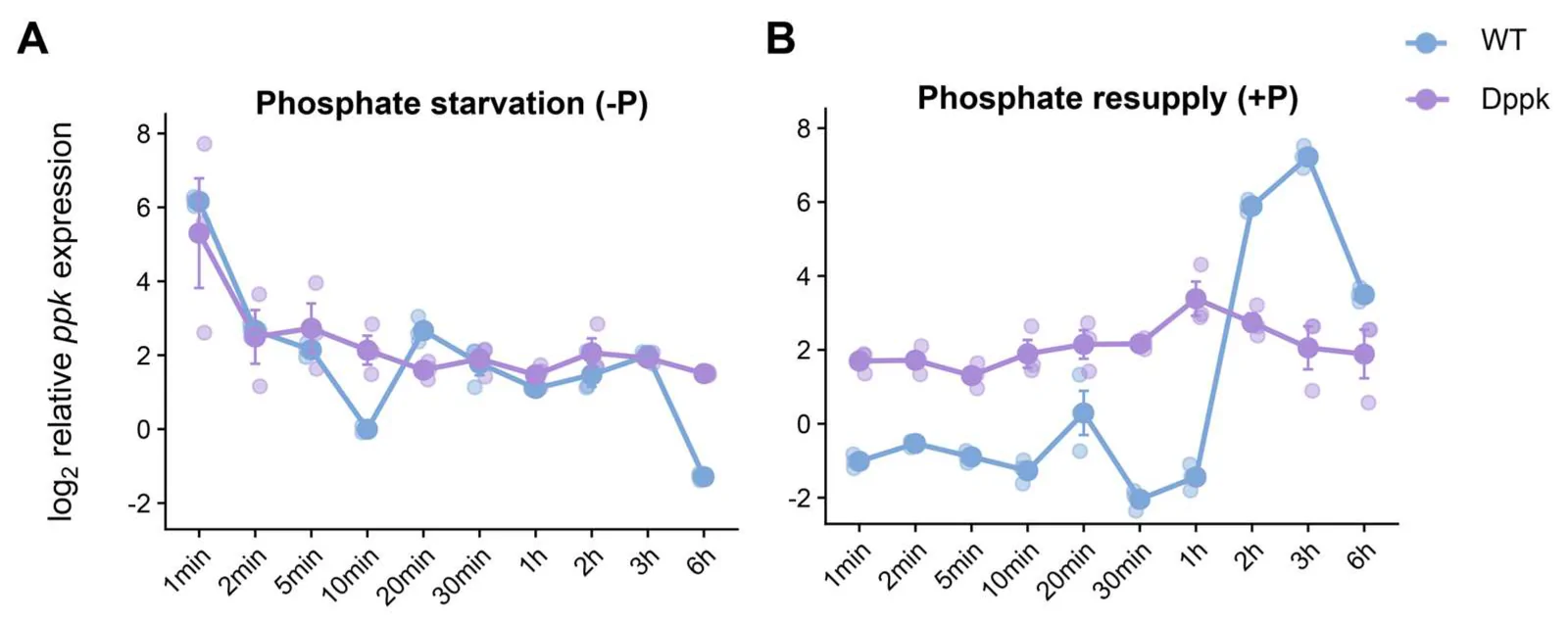
Strain-dependent temporal dynamics of *ppk* expression during phosphate fluctuation. (**A**) Time-course qPCR analysis of ppk expression in WT and Dppk after transfer to phosphate-free medium (−P). (**B**) Time-course qPCR analysis after phosphate resupply (+P). Relative expression was calculated against the corresponding initial condition for each strain.

To characterize the early response of *ppk* to phosphate transitions, its expression was monitored in WT and Dppk by RT-qPCR at closely spaced time points during phosphate starvation and subsequent phosphate resupply. In WT, native *ppk* expression changed rapidly within the first few minutes after transfer to phosphate-free medium, followed by pronounced fluctuations and a decline at later time points. By comparison, engineered *ppk* expression in Dppk showed smaller fluctuations after the initial transition and remained comparatively stable over most of the phosphate-starvation phase (Figure 2A).

The two strains also differed after phosphate resupply. In WT, *ppk* expression remained relatively low during the early resupply phase and then increased markedly, reaching a distinct peak at approximately 2–3 h after phosphate addition. Dppk exhibited a lower-amplitude response and lacked the pronounced late induction observed in WT (Figure 2B). These patterns indicate that native *ppk* expression in WT was highly responsive to phosphate transitions, whereas the engineered *ppk*-expression module in Dppk followed a more attenuated temporal pattern.

### 3.3. WT and Dppk Follow Distinct Global Transcriptomic Trajectories During Phosphate Fluctuation

**Figure 3:**
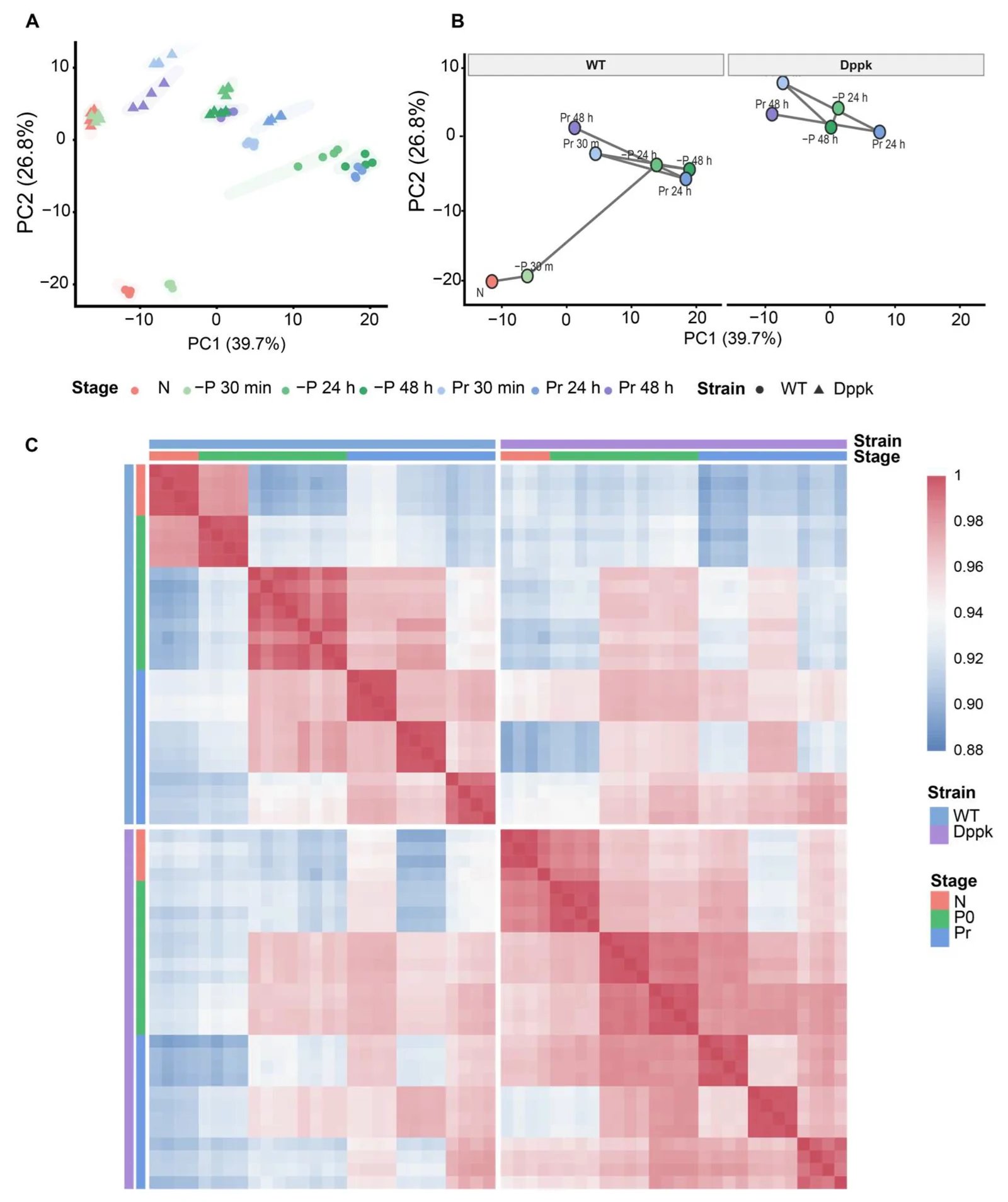
Global transcriptomic trajectories and sample correlations during phosphate fluctuation. (**A**) Principal component analysis (PCA) of VST-transformed RNA-seq expression profiles from WT and Dppk across the phosphate fluctuation series. Colors indicate experimental stages and symbols distinguish the two strains. Shaded regions are visual guides indicating clustering of biological replicates within each strain and stage. PC1 and PC2 explain 39.7% and 26.8% of the total variance, respectively. (**B**) Temporal trajectories of PCA centroids for WT and Dppk. Centroids represent the mean PC1 and PC2 coordinates of the four biological replicates at each stage and are connected in temporal order from N through phosphate starvation and subsequent resupply. (**C**) Pearson correlation heatmap of the 56 RNA-seq samples based on VST-transformed expression profiles. Annotation bars indicate strain and experimental stage, and the color scale represents the Pearson correlation coefficient.

To assess the global transcriptional responses of WT and Dppk to phosphate fluctuation, principal component analysis (PCA) was performed using variance-stabilized RNA-seq count data from all sampling stages. The first and second principal components explained 39.7% and 26.8% of the total variance, respectively. WT and Dppk occupied distinct regions of the PCA space even under the initial normal-phosphate condition, indicating different basal transcriptional states. In WT, the transcriptomic state shifted markedly from the normal-phosphate condition during prolonged phosphate starvation and changed further after phosphate resupply. Across the treatment series, both strains showed stage-dependent shifts but followed clearly different trajectories (Figure 3A). Biological replicates generally clustered closely within each strain–stage group, supporting the consistency of the RNA-seq dataset.

Sample-level Pearson correlation analysis based on the variance-stabilized expression matrix further supported the strain- and stage-dependent organization of the transcriptomes. Replicates within the same experimental group showed high similarity, whereas broader correlation patterns differed between WT and Dppk and changed across phosphate-starvation and resupply stages (Figure 3C). Together, the PCA and correlation analyses indicate that phosphate fluctuation was accompanied by distinct temporal restructuring of the transcriptome in WT and Dppk, superimposed on transcriptional differences already present under the normal-phosphate condition.

### 3.4. DEG Dynamics Reveal Strain-Dependent Phosphate-Response Patterns

**Figure 4:**
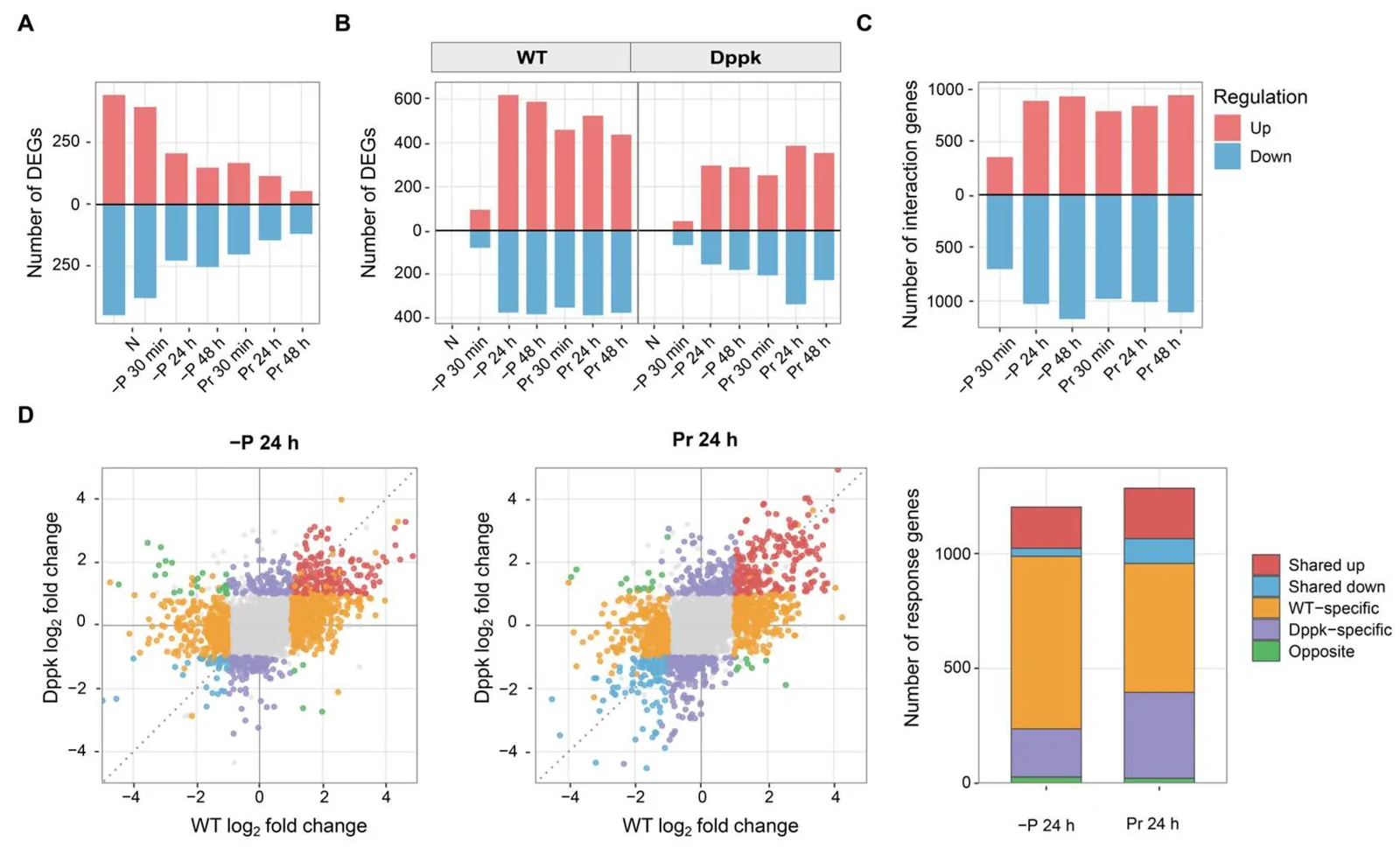
Temporal differential-expression patterns and response concordance of WT and Dppk during phosphate fluctuation. (**A**) Numbers of DEGs between Dppk and WT at each matched experimental stage. DEGs were defined using an adjusted *p* value < 0.05 and |log_2_ fold change| ≥ 1. (**B**) Numbers of phosphate-responsive DEGs within WT and Dppk, with each phosphate-starvation or resupply stage compared with the corresponding normal-phosphate condition (N) of the same strain. (**C**) Numbers of genes showing significant strain × stage interaction effects at each treatment stage relative to N. Positive and negative interactions indicate the sign of the interaction effect and do not represent conventional up or downregulation. (**D**) Response concordance between WT and Dppk at −P 24 h and Pr 24 h. Scatterplots compare within-strain log_2_ fold changes, and the stacked bars summarize genes classified as shared upregulated, shared downregulated, WT-specific, Dppk-specific, or opposite responses.

To characterize transcriptomic differences between WT and Dppk during phosphate fluctuation, differentially expressed genes (DEGs) were first identified between the two strains at each matched sampling stage. Numerous DEGs were already detected under the normal-phosphate condition, consistent with the distinct basal transcriptional states revealed by PCA. The number of DEGs between Dppk and WT subsequently varied across the phosphate-starvation and resupply series, while strain-associated differences remained detectable at all sampling stages (Figure 4A).

Phosphate-responsive DEGs were then identified within each strain by comparing each treatment stage with the corresponding normal-phosphate condition. In WT, the number of responsive DEGs increased markedly after 24 and 48 h of phosphate starvation and remained high after phosphate resupply. Dppk also showed substantial transcriptional responses across the treatment series but had fewer DEGs than WT at several corresponding stages, particularly during prolonged phosphate starvation (Figure 4B).

A strain × stage interaction analysis was further performed to directly test differences in temporal response between WT and Dppk while accounting for repeated sampling of the same biological cultures. The overall likelihood-ratio test identified 2942 genes with a significant strain-dependent temporal response (FDR < 0.05). Stage-specific interaction analyses detected significant interaction effects at all six treatment stages, involving 1056–2098 genes per stage. The fewest interaction genes were detected at −P 30 min, whereas larger numbers were observed at the later phosphate-starvation and resupply stages (Figure 4C).

Gene-wise response concordance was examined at −P 24 h and Pr 24 h. At both stages, shared upregulated and shared downregulated genes were detected together with WT-specific, Dppk-specific, and opposite-direction response groups. WT-specific genes represented a large proportion of the response genes at both stages, whereas the number of Dppk-specific genes was greater at Pr 24 h than at −P 24 h. Opposite-direction responses accounted for a relatively small fraction of the classified response genes (Figure 4D).

### 3.5. Functonal Enrichment Highlights Photosynthetic, Phosphate-, and Nitrogen-Related Transcriptional Responses

**Figure 5:**
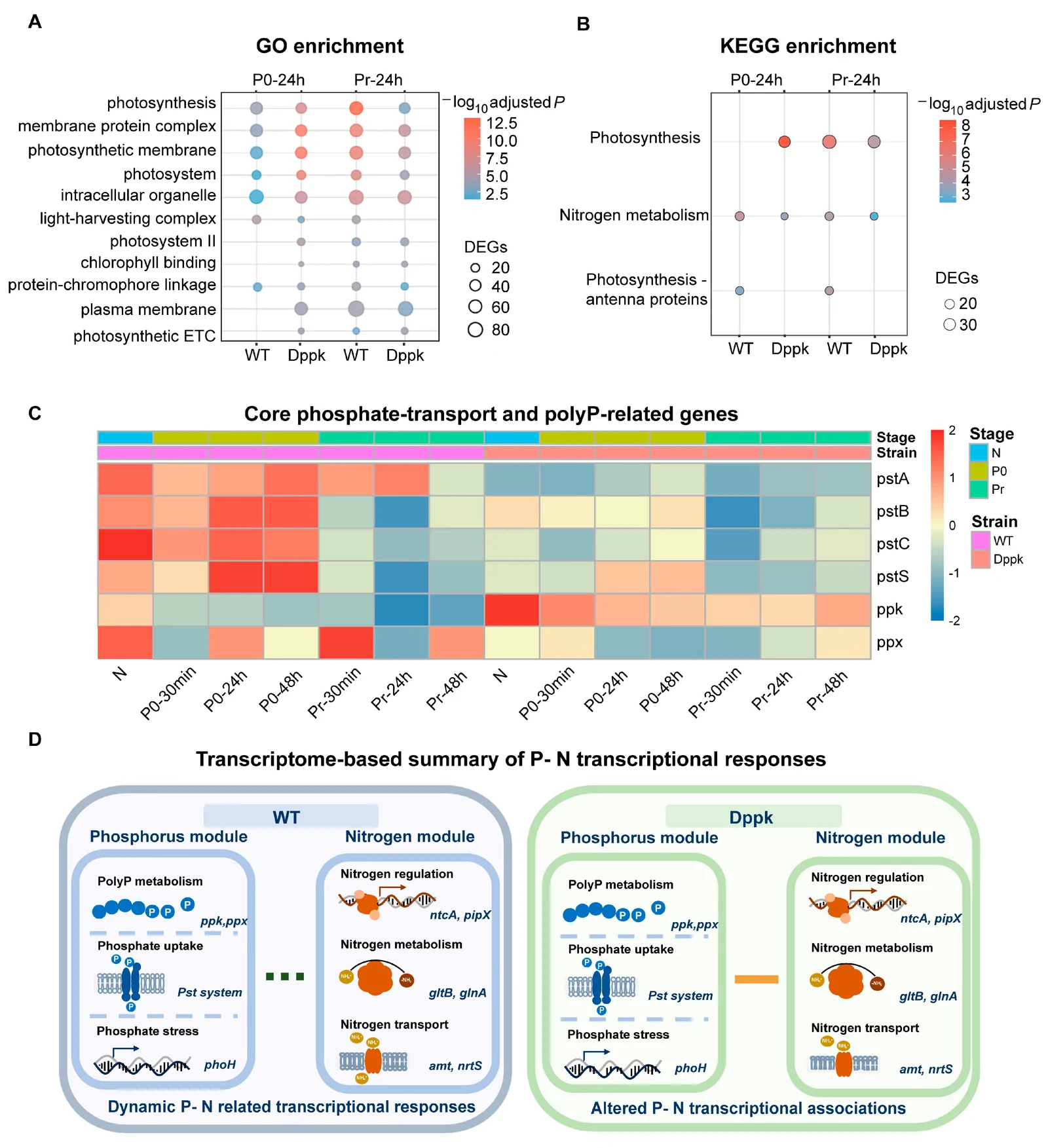
Functional enrichment and phosphorus- and nitrogen-related transcriptional responses during phosphate fluctuation. (**A**) Representative nonredundant GO terms enriched in WT and Dppk at −P 24 h and Pr 24 h. Redundancy among significant GO terms was reduced according to overlap of contributing gene sets. (**B**) Significant KEGG pathway enriched in WT and Dppk at the same stages. In (**A**,**B**), dot size represents the number of contributing DEGs and color indicates −log_10_ (adjusted *p* value). (**C**) Row-scaled Z-score heatmap showing temporal expression patterns of the phosphate transport genes *pstA*, *pstB*, *pstC*, and *pstS* and the polyP metabolism genes *ppk* and *ppx*. Z-scores represent relative temporal variation within each gene. (**D**) Transcriptome-based schematic summary of phosphorus- and nitrogen-related responses in WT and Dppk during phosphate fluctuation. Representative genes and functional categories illustrate differences in phosphate transport, polyP-related transcription, photosynthesis-associated responses, and nitrogen-related transcriptional patterns. The P–N component represents transcriptional associations rather than experimentally demonstrated regulatory interactions or physiological coordination.

To identify the biological functions associated with phosphate fluctuation, GO and KEGG enrichment analyses were performed at the representative −P 24 h and Pr 24 h stages. These stages corresponded to those used for response concordance analysis in Figure 4. GO enrichment identified multiple functions associated with photosynthetic organization and activity. Following redundancy reduction based on overlap among the contributing gene sets, the representative terms included photosynthesis, photosynthetic membrane, photosystem, photosystem II, light-harvesting complex, protein–chromophore linkage, photosynthetic electron transport chain, membrane protein complex, plasma membrane, and intracellular organelle (Figure 5A). These results indicate that cellular structures and processes involved in photosynthesis constituted a prominent component of the transcriptional response to phosphate fluctuation.

KEGG enrichment analysis showed a similar functional pattern. The major enriched pathways included photosynthesis, photosynthesis antenna proteins, and nitrogen metabolism (Figure 5B). Photosynthesis was enriched in Dppk during phosphate starvation and in both strains after phosphate resupply, whereas photosynthesis antenna proteins were detected mainly in WT. Nitrogen metabolism was enriched in several comparisons, showing that phosphate fluctuation was accompanied by transcriptional changes in nitrogen-related processes.

The expression patterns of selected phosphate-transport and polyP-related genes were subsequently examined. The phosphate-transport genes *pstA*, *pstB*, *pstC*, and *pstS*, together with the polyP metabolism-related genes *ppk* and *ppx*, displayed strain-dependent temporal patterns during phosphate starvation and phosphate resupply (Figure 5C). In WT, several *pst* genes showed pronounced temporal changes during phosphate starvation, whereas Dppk exhibited distinct expression trajectories for the pst genes and altered temporal patterns of *ppk* and *ppx*. Because each gene was scaled independently in the heatmap, the color variation represents relative temporal changes within individual genes rather than absolute expression differences among genes.

Consistent with the enrichment of nitrogen metabolism, genes involved in nitrogen regulation, assimilation, and transport also showed expression patterns that varied with strain and stage (Figure S2A). Summaries at the module level revealed distinct temporal trajectories for nitrogen regulation, nitrogen assimilation, ammonium transport, nitrate assimilation, and utilization of alternative nitrogen sources in WT and Dppk (Figure S2B). In WT, the nitrogen-regulation and ammonium-transport modules increased during prolonged phosphate starvation, whereas Dppk showed different response amplitudes and recovery-stage patterns. The nitrogen-assimilation module also differed between the two strains, particularly during phosphate resupply. An exploratory P–N association analysis further revealed differences in the correlation patterns of selected phosphorus- and nitrogen-related genes between WT and Dppk (Figure S2B). For visualization, the network retained P–N gene pairs with |Δr| ≥ 0.5, where Δr = rDppk − rWT, thereby summarizing pairs with comparatively large differences in correlation coefficients between the two strains (Figure 5D).

## 4. Discussion

### 4.1. Modified ppk Expression Reshapes the Temporal Response to Phosphate Fluctuation

A prominent gene-level feature of the present study was the difference in the temporal pattern of *ppk* expression between WT and Dppk during phosphate starvation and subsequent resupply. The densely sampled RT-qPCR series resolved rapid expression changes that were not captured by the longer RNA-seq sampling intervals. In WT, ppk expression changed markedly across successive phases of phosphate withdrawal and resupply, whereas Dppk showed comparatively more moderate fluctuations over the same treatment series. These observations indicate that the two strains differed not only in ppk expression level but also in the temporal organization of the transcriptional response.

The delayed increase in *ppk* expression observed in WT after phosphate resupply further indicates that recovery did not simply represent an immediate reversal of the starvation response. In PCC 7002, phosphorus deficiency affects phosphate acquisition, photosynthesis, ribosomal functions, motility, and central metabolism, while phosphate restoration is accompanied by a distinct transcriptional transition [23]. In *Prochlorococcus* MED4, uptake physiology and gene expression likewise depend on the duration and history of phosphorus stress [14,40]. Rapid polyP accumulation after phosphate resupply has also been observed in *Synechocystis* and *Nostoc*, while elements of the starvation-acclimated state may persist during early recovery [16,17]. In this context, the late *ppk* increase in WT may reflect a recovery-associated regulatory state rather than a simple response to the external phosphate concentration. This is consistent with the stage-dependent expression pattern observed across the starvation–resupply sequence.

The temporal pattern in Dppk differed from that in WT, particularly in the absence of the pronounced late increase observed after phosphate resupply. In Dppk, the endogenous *ppk* locus was replaced while a P*_cpcBA_*-driven *ppk* copy was retained, thereby placing *ppk* expression under a different regulatory architecture. Engineered expression systems can reduce dependence on selected native regulatory inputs but remain subject to the broader physiological state of the host [19,41]. Promoter activity, RNA turnover, transcriptional capacity, resource availability, and cellular energy status may therefore continue to influence expression from the engineered cassette. The expression pattern observed in Dppk is consequently consistent with reduced dependence on the native phosphate-responsive regulatory context, although the present comparison cannot assign the observed difference exclusively to a specific regulatory element.

These transcriptional differences should not be interpreted as direct measurements of PPK activity or polyP synthesis. Cyanobacterial polyP accumulation depends on external phosphate availability, growth state, and the balance among polyP synthesis, degradation, and cellular phosphorus demand, and therefore cannot be inferred from *ppk* transcript abundance alone [15,18,42]. The present data establish that WT and Dppk displayed distinct temporal patterns of *ppk* transcription during phosphate starvation and resupply. Whether these gene-level differences were accompanied by broader changes in the transcriptional response to phosphate fluctuation was further examined at the whole-transcriptome level.

### 4.2. Dppk Exhibits a Distinct Temporal Transcriptional Response to Phosphate Transitions

Global transcriptomic analyses showed that WT and Dppk followed distinct trajectories during phosphate starvation and subsequent resupply. Although both strains underwent pronounced stage-dependent transcriptional changes, the strain × stage interaction analysis identified widespread differences in their temporal responses, indicating that the distinction between the two strains extended beyond their basal transcriptional states. Within-strain analyses further showed that Dppk had fewer conventionally defined phosphate-responsive DEGs than WT at several stages. However, DEG number alone does not quantify response magnitude and is therefore interpreted here as a descriptive feature of the temporal response.

WT and Dppk were already transcriptionally distinct before phosphate withdrawal, which likely influenced their subsequent trajectories through the treatment series. The concordance analysis further showed that the two strains shared subsets of phosphate-responsive genes while also exhibiting substantial strain-specific and a smaller number of opposite-direction responses. Similar stage-dependent overlap and divergence have been reported in marine *Synechococcus* and *Prochlorococcus*, where early and prolonged phosphorus stress recruit partially distinct transcriptional programs [13,40]. Progressive development of phosphorus-stress responses has also been observed in *Crocosphaera*, emphasizing the importance of resolving acclimation over time rather than at a single endpoint [43]. These patterns are compatible with the multilayered cyanobacterial transcriptome, where environmentally responsive promoters and regulatory transcripts extend acclimation beyond a single canonical regulon [44]. Together, these observations indicate that the difference between WT and Dppk involved both the timing and composition of the phosphate-responsive transcriptome.

These transcriptional differences should not be equated with improved phosphate acclimation or stress tolerance. Phosphate uptake, polyP turnover, growth, and photosynthetic performance cannot be inferred directly from transcript abundance, and phosphorus acclimation also involves regulation at the protein and metabolic levels [45]. The present data therefore support distinct transcriptional response programs in WT and Dppk, while their physiological consequences require direct functional validation. The functional processes associated with these strain-dependent responses are examined below.

### 4.3. Phosphate Fluctuation Engages Photosynthetic and Phosphate-Homeostasis Transcriptional Programs

The functional analyses place the strain-dependent response within an integrated phosphate-energy framework. GO terms related to photosynthesis, photosynthetic and thylakoid membranes, photosystems, phycobilisomes, and light-harvesting complexes, together with KEGG enrichment of photosynthesis and antenna-protein pathways, represent different levels of the same cellular system rather than a collection of unrelated functions. In PCC 7002, phosphate limitation affects photosynthetic, ribosomal, motility, transport, and central metabolic processes, while phosphate restoration initiates broad transcriptional reorganization [23]. Proteomic and transcriptomic studies in other cyanobacteria likewise connect phosphate acclimation with changes in photosynthetic proteins and thylakoid-associated metabolism [45,46]. Phosphorus deficiency in *Raphidiopsis raciborskii* was shown to induce broad transcriptional changes involving photosynthetic electron transport and nutrient metabolism [47]. Phosphate deprivation can also drive replacement of phospholipids with phosphorus-free glycolipids, directly linking phosphorus conservation to photosynthetic membrane remodeling [48]. The convergence of these categories in the present study therefore indicates that phosphorus fluctuation was integrated with the transcriptional organization of energy capture and membrane-associated metabolism.

This interpretation is supported by the targeted phosphate-homeostasis genes. PstSCAB is an ATP-dependent high-affinity phosphate transport system, and its expression is regulated by phosphate-responsive systems such as SphS–SphR; distinct Pst systems can also differ in transport properties [11,12]. PCC 7002 similarly induces phosphate-transport genes during phosphorus deficiency, and disruption of selected transport-related genes compromises growth under low-phosphate conditions [23]. Against this background, the divergent *pstA*, *pstB*, *pstC*, and *pstS* trajectories in WT and Dppk support altered temporal coordination of phosphate acquisition. They do not, however, demonstrate differences in transporter affinity or phosphate uptake rate, which were not measured directly.

The lack of parallel behavior among *pst*, *ppk*, *ppx*, and *phoH* is equally informative. Phosphate uptake, incorporation into essential metabolites, storage as polyP, and subsequent remobilization need not occur simultaneously. Cyanobacterial polyP pools can accumulate rapidly after phosphate becomes available and decline as growth and biosynthetic demand increase [15,17]. Consistent with a temporally distinct remobilization program, *ppa* and *ppx* are transcriptionally induced during prolonged phosphate deprivation in PCC 6803 [49]. Genetic studies further show that the physiological contribution of polyP metabolism is strongly condition dependent: disruption of polyP-related functions alters stress acclimation in thermophilic *Synechococcus*, while impaired PPX-dependent polyP mobilization in PCC 6803 compromises survival and photosystem stability during phosphate starvation [49,50]. PPK1-dependent polyP accumulation likewise becomes physiologically important under selected nutrient-loss states rather than under all growth conditions [18]. The altered relationships among these genes in Dppk therefore are consistent with differences in phosphate homeostasis-associated transcriptional programs.

The photosynthetic enrichments can be interpreted within the same resource-allocation framework. Phosphate acquisition and cellular phosphorus metabolism are closely connected with ATP demand, biosynthesis, membrane organization, and photosynthetic energy metabolism, and perturbation of PPK has also been associated with changes in cellular energy status after phosphate re-addition [51]. Because the enrichment analysis included both upregulated and downregulated genes and no photosynthetic measurements were performed, these results indicate differences in photosynthesis-related transcription rather than enhanced or suppressed photosynthetic performance in Dppk.

Finally, the enrichment of nitrogen metabolism and the distinct temporal patterns of nitrogen-related modules indicated that the transcriptional response extended beyond phosphorus-specific functions, motivating an exploratory examination of P–N transcriptional associations.

### 4.4. Modified ppk Expression Architecture Is Associated with Altered Phosphorus–Nitrogen Transcriptional Association

Phosphorus and nitrogen responses in cyanobacteria are integrated with cellular carbon and energy status. The 2-oxoglutarate–NtcA and PII–PipX regulatory systems connect nitrogen assimilation and transport with metabolic and energetic signals [24,25,26]. While the direct NtcA regulon in PCC 6803 includes pstS, providing experimental evidence for transcriptional overlap between nitrogen status and phosphate acquisition [27]. Phosphorus availability has also been shown to interact with nitrogen-related and energy-allocation programs in other cyanobacteria [52,53]. These established connections provide a biological basis for examining P- and N-related transcriptional responses together, without implying direct regulation of nitrogen-responsive genes by PPK.

Within this framework, nitrogen-associated genes showed distinct temporal responses in WT and Dppk. Genes involved in nitrogen regulation, GS–GOGAT-dependent assimilation, ammonium transport, nitrate-related functions, and alternative nitrogen sources differed in the timing and persistence of their responses across phosphate starvation and resupply (Supplementary Figure S2A). These modules did not shift uniformly in one direction; rather, the data indicate differences in the temporal distribution of nitrogen-related transcriptional responses between the two strains. Such responses are consistent with the broader nutrient-responsive plasticity previously reported for PCC 7002 [23].

Exploratory correlation analysis further showed that selected phosphorus- and nitrogen-related genes differed in their patterns of transcriptional co-variation between WT and Dppk. Correlations were calculated separately across the complete sample-level phosphate-transition series, and differences were expressed as Δr = r_Dppk − r_WT. Individual P–N pairs showed both positive and negative Δr values, indicating that some correlations were higher in Dppk whereas others were lower (Supplementary Figure S2B). Because the same biological cultures were repeatedly sampled across the time course, these observations were not statistically independent; the Δr analysis is therefore interpreted only as a descriptive assessment of strain-dependent transcriptional associations.

Figure 5D summarizes these phosphorus- and nitrogen-related expression patterns in the context of the broader phosphate response. The P–N component represents transcriptional associations observed during phosphate fluctuation rather than a mechanistic model of phosphorus–nitrogen regulation. PolyP dynamics, phosphate uptake, intracellular phosphorus and nitrogen status, nitrogen-assimilation fluxes, and corresponding physiological consequences were not directly measured. These properties cannot be inferred from transcript abundance alone, and physiological and metabolic validation will be required to determine whether the observed transcriptional associations translate into functional changes in P–N homeostasis.

## 5. Conclusions

This study characterized the temporal transcriptional responses of WT and Dppk, an engineered strain with a modified *ppk* expression architecture, during phosphate starvation and subsequent phosphate resupply. Compared with WT, Dppk exhibited more moderate ppk expression dynamics and distinct temporal transcriptional patterns while retaining a shared core phosphate-responsive program. The strain-associated responses involved phosphate transport, polyphosphate metabolism, photosynthesis-related functions, and selected nitrogen-related processes. In addition, phosphorus- and nitrogen-related genes showed distinct transcriptional association patterns between the two strains, suggesting that modification of the *ppk* expression architecture is accompanied by broader changes in the organization of phosphate-responsive transcription. These findings provide a basis for further investigating how engineered *ppk* expression influences cellular acclimation to fluctuating phosphate availability. However, because only one engineered Dppk isolate was examined, potential clone-specific or secondary genetic effects cannot be completely excluded and should be addressed in future studies using independently reconstructed clones or genome-level verification. From an applied perspective, such engineering strategies may have potential relevance to cyanobacterial nutrient management and phosphorus recovery under variable nutrient conditions. However, direct physiological measurements of polyP dynamics, nutrient fluxes, and process-scale performance will be required to determine whether these transcriptional differences translate into improved functional or industrial performance.

## Data Availability

The raw RNA-sequencing data generated in this study have been submitted to the NCBI Sequence Read Archive (SRA) under BioProject accession number PRJNA1504822 and will be publicly released upon publication. The processed data supporting the findings of this study are provided within the article and its Supplementary Materials.

## Supplementary Materials

The following supporting information can be downloaded at: https://www.mdpi.com/article/10.3390/microorganisms14091997/s1, Table S1: Composition of A+ medium; Table S2: Primers used for Dppk constructions and RT-qPCR; Table S3: Sample metadata and RNA-seq quality-control metrics; Figure S1: Representative melting curves for RT-qPCR amplification of 16S rRNA; Figure S2: Temporal expression patterns of nitrogen-associated genes and exploratory phosphorus–nitrogen transcriptional associations during phosphate fluctuation. Supplementary Data S1: RNA-seq gene-level read-count matrix; Supplementary Data S2: Complete differential-expression results; Supplementary Data S3: Strain × stage interaction-analysis results; Supplementary Data S4: Complete GO enrichment results; Supplementary Data S5: Complete KEGG enrichment results; Supplementary Data S6: Phosphorus–nitrogen transcriptional-analysis data.
