# Supplementary material for: Modified ppk Expression Alters Temporal Transcriptional and Phosphorus–Nitrogen Responses to Phosphate Fluctuation in Synechococcus sp. PCC 7002
Source: Microorganisms. 2026 Sep 9;14(9):1997. doi: 10.3390/microorganisms14091997 (PMC13609420; doi:10.3390/microorganisms14091997)
Supplement: Supplementary file 1 [file microorganisms-14-01997-s001.zip › microorganisms-4504339-supplementary.pdf]

Supplementary Materials

Modified *ppk* Expression Alters Temporal Transcriptional and Phosphorus–Nitrogen Responses to Phosphate Fluctuation in *Synechococcus* sp. PCC 7002

Sijia Shen [1,2], Enci Ye [1,2], Tao Li [1,3] and Zi Ye [1,3,4,*]

1 Institute of Hydrobiology, Chinese Academy of Sciences, Wuhan 430072, China; shensijia@ihb.ac.cn (S.S.); yeenci@ihb.ac.cn (E.Y.); litao@ihb.ac.cn (T.L.)
2 University of Chinese Academy of Sciences, Beijing 100049, China
3 Key Laboratory of Algal Biology, Chinese Academy of Sciences, Wuhan 430072, China
4 State Key Laboratory of Lake and Watershed Science for Water Security, Institute of Hydrobiology, Chinese Academy of Sciences, Wuhan 430072, China
* Correspondence: yezi@ihb.ac.cn

Table S1. Composition of A+ medium

Substance	Concentration	Stock Solution	V to 1L Culture
$NaNO_3$	1 g/L	/	/
NaCl	18 g/L	/	/
$MgSO4·7H_2O$	5 g/L	50 ×	20 mL
Tris-HCl（pH8.2）	1 g/L	100 ×	10 mL
KCl	600 mg/L	500 ×	2 mL
*$CaCl_2$	270 mg/L	1000 ×	1 mL
*KH_2PO_4	50 mg/L	1000 ×	1 mL
EDTA	30 mg/L	500 ×	2 mL
*Na_2CO_3	20 mg/L	1000 ×	1 mL
$FeCl_3·6H_2O$	3.89 mg/L	1000 ×	1 mL
**H_3BO_3	2.86 mg/L	500 ×	
**$MnCl·4H_2O$	1.81 mg/L	500 ×	
**$ZnSO_4·7H_2O$	0.22 mg/L	500 ×	
**$Na_2MoO_4·2H_2O$	0.39 mg/L	500 ×	
**$CuSO_4·5H_2O$	0.1 mg/L	500 ×	
**Co（NO_3）$_2·6H_2O$	0.05 mg/L	500 ×	
*Vitamin B12	4 μg/L	1000 ×	

Note:* Added separately after sterilization in a clean bench; ** Prepare as A5 solution before use.

Table S2. Primers used for *Dppk* constructions and RT-qPCR.

Name	Sequence	Purpose
Up-kan-F	ATCCTCTGCATAAAAAGTATAAGAAGGTGTTGCTGACTCA	Kanamycin resistance cassette
Down-kan-R	TGAAACGCGCGACAACGAAAATTAGAAAAACTCATCGAGCA	Kanamycin resistance cassette
Up-ppk-R	TGAGTCAGCAACACCTTCTTATACTTTTTATGCAGAGGAT	*ppk* upstream homologous arm
Up-ppk-F	TTCGAGCTCGGTACCCGGGGAGGGCGAGACAAATAAAGGGA	*ppk* upstream homologous arm
Down-ppk-F	TGCTCGATGAGTTTTTCTAATTTTCGTTGTCGCGCGTTTCA	*ppk* downstream homologous arm
Down-ppk-R	CTGCAGGTCGACTCTAGAGGATTTATGGCTCGACCGAATCA	*ppk* downstream homologous arm
vector-R	TCCCTTTATTTGTCTCGCCCTCCCCGGGTACCGAGCTCGAA	Vector backbone amplification
vector-F	TGATTCGGTCGAGCCATAAATCCTCTAGAGTCGACCTGCAG	Vector backbone amplification
ppk NdeI	CAACATATGTCCTCTGCGAAAACCGC	*ppk* amplification
ppk-Bgl II	TCAAATCTAATTATTTTAGAGTTCATTGAATTAATCTCCTACTTGACTTTATGAGT	*ppk* amplification
16s-F	CGGGTTTGATGAGATTCGCTTGC	RT-qPCR
16s-R	AGTTGGGCACTCTAGGGAGACTG	RT-qPCR
ppk-F	AGCATATTTTCCCCGTCCTG	RT-qPCR
ppk-R	GGCTGAGGTTAGAAATGTGGG	RT-qPCR

Table S3. Sample metadata and RNA-seq quality-control metrics

Sample ID	Strain	Culture ID	Stage	Raw reads	GC rate	Q30_rate	Filtered reads	Mapping rate	Unmapped rate
WT_N_1	WT	WT_C1	N	17280732	49.21%	95.56%	16925968	96.20%	3.80%
WT_N_2	WT	WT_C2	N	15704632	48.85%	96.10%	15472498	97.32%	2.68%
WT_N_3	WT	WT_C3	N	15069736	49.12%	96.03%	14873438	96.82%	3.18%
WT_N_4	WT	WT_C4	N	14918426	49.13%	96.07%	14768390	97.33%	2.67%
WT_P0_30min_1	WT	WT_C1	P0_30min	15822988	48.02%	95.62%	15652666	94.80%	5.20%
WT_P0_30min_2	WT	WT_C2	P0_30min	17682850	47.80%	96.16%	17428288	96.10%	3.90%
WT_P0_30min_3	WT	WT_C3	P0_30min	16328268	47.87%	96.14%	16133594	95.16%	4.84%
WT_P0_30min_4	WT	WT_C4	P0_30min	16044516	47.50%	95.83%	15765310	93.09%	6.91%
WT_P0_24h_1	WT	WT_C1	P0_24h	16137398	48.06%	95.66%	15889930	94.40%	5.60%
WT_P0_24h_2	WT	WT_C2	P0_24h	14350166	48.41%	95.66%	14069342	95.29%	4.71%
WT_P0_24h_3	WT	WT_C3	P0_24h	16013636	47.87%	95.36%	15778402	92.55%	7.45%
WT_P0_24h_4	WT	WT_C4	P0_24h	15094138	47.88%	95.55%	14916864	93.29%	6.71%
WT_P0_48h_1	WT	WT_C1	P0_48h	15547220	48.48%	95.63%	15352534	94.22%	5.78%
WT_P0_48h_2	WT	WT_C2	P0_48h	13099082	48.76%	95.85%	12985276	95.49%	4.51%
WT_P0_48h_3	WT	WT_C3	P0_48h	13467784	48.45%	95.79%	13333858	94.78%	5.22%
WT_P0_48h_4	WT	WT_C4	P0_48h	14537950	48.40%	95.67%	14354522	94.86%	5.14%
WT_Pr_30min_1	WT	WT_C1	Pr_30min	21649072	49.74%	95.94%	21372762	93.42%	6.58%
WT_Pr_30min_2	WT	WT_C2	Pr_30min	15745604	48.89%	96.04%	15462944	93.18%	6.82%
WT_Pr_30min_3	WT	WT_C3	Pr_30min	18276958	48.82%	95.75%	17966064	92.77%	7.23%
WT_Pr_30min_4	WT	WT_C4	Pr_30min	19243298	49.06%	95.94%	18998188	93.31%	6.69%
WT_Pr_24h_1	WT	WT_C1	Pr_24h	17651774	48.51%	96.05%	17392840	92.57%	7.43%
WT_Pr_24h_2	WT	WT_C2	Pr_24h	19245802	48.26%	96.09%	18996872	94.20%	5.80%
WT_Pr_24h_3	WT	WT_C3	Pr_24h	18696396	48.27%	96.00%	18360010	93.19%	6.81%

WT_Pr_24h_4	WT	WT_C4	Pr_24h	20652422	49.43%	96.03%	20316558	93.12%	6.88%
WT_Pr_48h_1	WT	WT_C1	Pr_48h	22212062	51.92%	95.65%	21532082	93.73%	6.27%
WT_Pr_48h_2	WT	WT_C2	Pr_48h	20973304	50.45%	95.96%	20501100	92.58%	7.42%
WT_Pr_48h_3	WT	WT_C3	Pr_48h	21306344	49.50%	96.02%	21000000	92.14%	7.86%
WT_Pr_48h_4	WT	WT_C4	Pr_48h	19016264	50.03%	95.90%	18736346	92.20%	7.80%
Dppk_N_1	Dppk	Dppk_C1	N	12553302	49.61%	95.62%	12464506	94.44%	5.56%
Dppk_N_2	Dppk	Dppk_C2	N	16423418	49.05%	96.04%	16257220	97.05%	2.95%
Dppk_N_3	Dppk	Dppk_C3	N	14352966	49.45%	95.84%	14253814	95.99%	4.01%
Dppk_N_4	Dppk	Dppk_C4	N	11320918	48.22%	96.09%	11232038	94.90%	5.10%
Dppk_P0_30min_1	Dppk	Dppk_C1	P0_30min	16412280	48.10%	95.94%	16249658	93.71%	6.29%
Dppk_P0_30min_2	Dppk	Dppk_C2	P0_30min	14787968	48.40%	96.12%	14606610	94.82%	5.18%
Dppk_P0_30min_3	Dppk	Dppk_C3	P0_30min	15411116	48.01%	96.00%	15261854	95.10%	4.90%
Dppk_P0_30min_4	Dppk	Dppk_C4	P0_30min	13848448	47.77%	95.87%	13642090	93.61%	6.39%
Dppk_P0_24h_1	Dppk	Dppk_C1	P0_24h	13457638	48.34%	96.07%	13254678	92.42%	7.58%
Dppk_P0_24h_2	Dppk	Dppk_C2	P0_24h	14395270	49.08%	95.94%	14276048	91.43%	8.57%
Dppk_P0_24h_3	Dppk	Dppk_C3	P0_24h	12998486	48.77%	95.90%	12766110	92.52%	7.48%
Dppk_P0_24h_4	Dppk	Dppk_C4	P0_24h	13430556	48.48%	95.80%	13227226	90.91%	9.09%
Dppk_P0_48h_1	Dppk	Dppk_C1	P0_48h	15361486	48.65%	95.91%	15208054	92.17%	7.83%
Dppk_P0_48h_2	Dppk	Dppk_C2	P0_48h	12444464	48.28%	95.89%	12279800	93.35%	6.65%
Dppk_P0_48h_3	Dppk	Dppk_C3	P0_48h	13318840	48.63%	95.95%	13175610	93.67%	6.33%
Dppk_P0_48h_4	Dppk	Dppk_C4	P0_48h	16141650	48.17%	96.04%	15972010	92.73%	7.27%
Dppk_Pr_30min_1	Dppk	Dppk_C1	Pr_30min	14638922	48.27%	96.17%	14451090	93.71%	6.29%
Dppk_Pr_30min_2	Dppk	Dppk_C2	Pr_30min	15923396	48.37%	96.18%	15749994	93.81%	6.19%
Dppk_Pr_30min_3	Dppk	Dppk_C3	Pr_30min	14263936	48.76%	96.03%	14123202	92.79%	7.21%
Dppk_Pr_30min_4	Dppk	Dppk_C4	Pr_30min	13283678	48.49%	96.02%	13121038	93.50%	6.50%
Dppk_Pr_24h_1	Dppk	Dppk_C1	Pr_24h	14883836	48.81%	96.11%	14740440	94.39%	5.61%

Dppk_Pr_24h_2	Dppk	Dppk_C2	Pr_24h	13928584	48.82%	96.01%	13740488	94.23%	5.77%
Dppk_Pr_24h_3	Dppk	Dppk_C3	Pr_24h	13357814	48.84%	95.96%	13159134	94.60%	5.40%
Dppk_Pr_24h_4	Dppk	Dppk_C4	Pr_24h	14637462	48.71%	95.90%	14394428	94.80%	5.20%
Dppk_Pr_48h_1	Dppk	Dppk_C1	Pr_48h	13585236	48.80%	96.03%	13419216	94.56%	5.44%
Dppk_Pr_48h_2	Dppk	Dppk_C2	Pr_48h	12755292	48.73%	96.12%	12514134	94.35%	5.65%
Dppk_Pr_48h_3	Dppk	Dppk_C3	Pr_48h	13701752	48.36%	95.89%	13462112	94.91%	5.09%
Dppk_Pr_48h_4	Dppk	Dppk_C4	Pr_48h	15009888	48.74%	96.00%	14825628	94.70%	5.30%

Note：Raw and clean read counts, GC content, Q30 values, and genome-mapping rates are shown for each RNA-seq library. Culture ID denotes the biological culture that was repeatedly sampled across the seven experimental stages. All 56 libraries were retained for downstream analyses.

Supplementary Figures

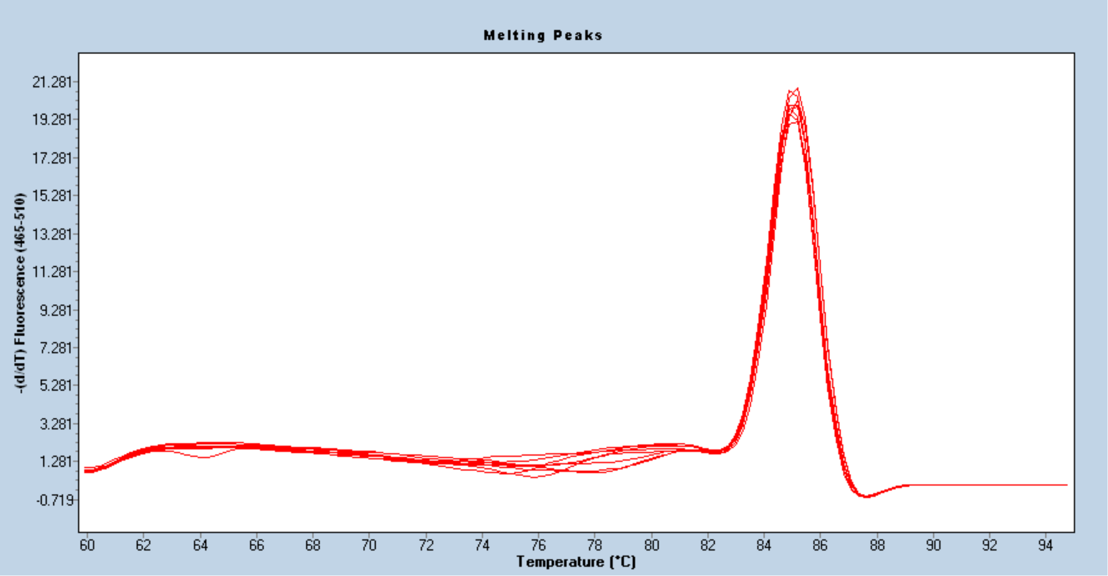

Figure S1

Representative melting curves for RT-qPCR amplification of 16S rRNA.

A

B

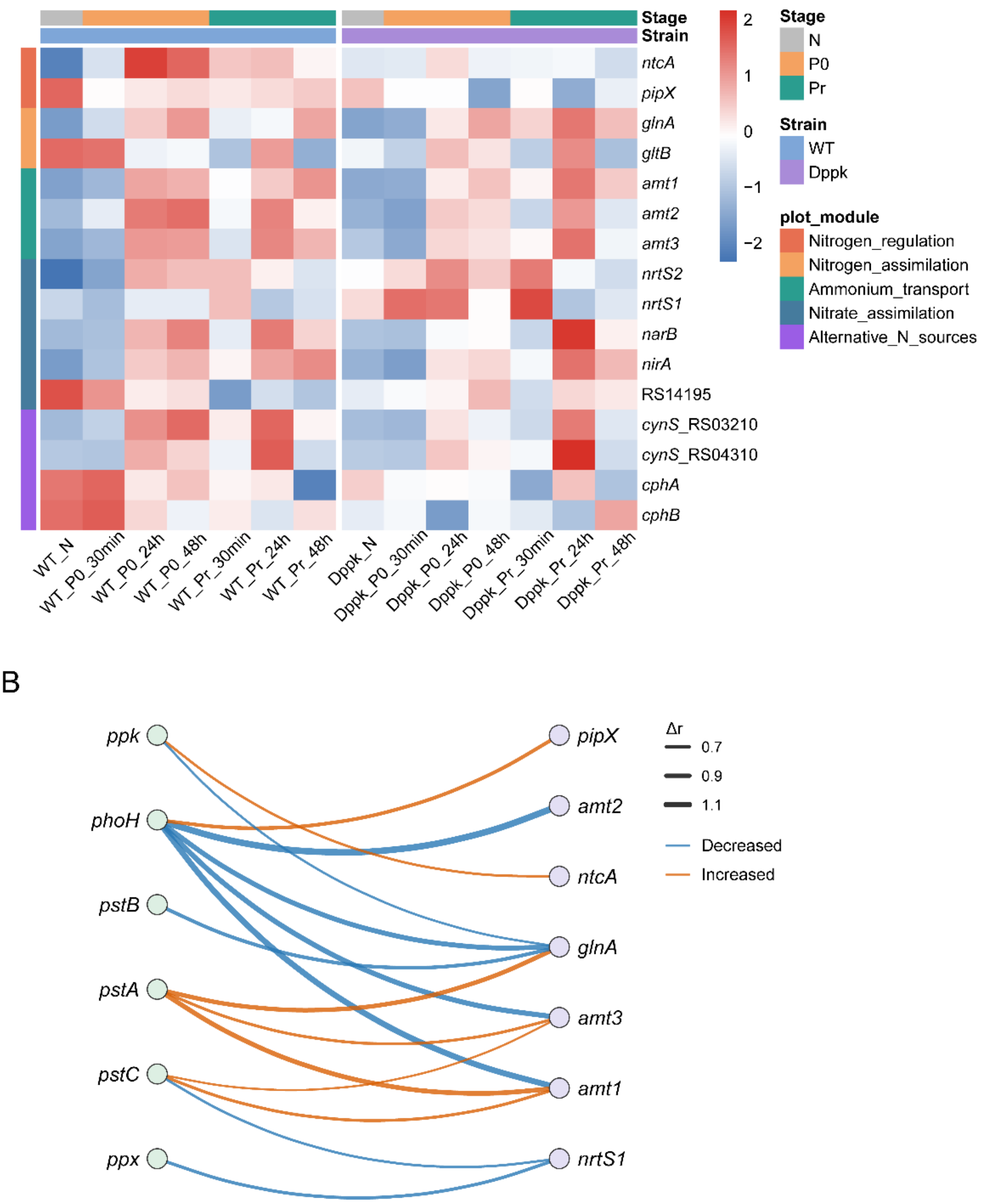

Figure S2

Temporal expression patterns of nitrogen-associated genes and exploratory phosphorus–nitrogen transcriptional associations during phosphate fluctuation.

(A) Heatmap showing the temporal expression patterns of selected nitrogen-associated genes in WT and Dppk during phosphate starvation and resupply. FPKM values were log2-transformed as log2(FPKM + 1), averaged across the four biological cultures within each strain–stage group, and standardized for each gene across the 14 strain–stage groups by row-wise Z-score transformation. Genes were grouped according to nitrogen regulation, nitrogen assimilation, ammonium transport, nitrate-related functions, and alternative nitrogen-source or storage-related functions.

(B) Exploratory differences in transcriptional associations between selected phosphorus- and nitrogen-related genes. Pearson correlation coefficients were calculated separately for

WT and Dppk using the 28 sample-level expression profiles per strain, and differences were expressed as $\Delta r = r\_Dppk - r\_WT$. P–N gene pairs with $|\Delta r| \geq 0.5$ are shown for visualization. Orange and blue edges indicate increased and decreased correlation coefficients in Dppk relative to WT, respectively, and edge width represents $|\Delta r|$. Phosphorus-related genes are shown on the left and nitrogen-related genes on the right. Because samples from the same biological cultures were repeatedly collected across the time course, the correlation analysis is presented as an exploratory description of transcriptional associations rather than a formal test of regulatory interactions. N, normal-phosphate condition; P0, phosphate starvation; Pr, phosphate resupply.
